# Combining Users’ Needs With Health Behavior Models in Designing an Internet- and Mobile-Based Intervention for Physical Activity in Cardiac Rehabilitation

**DOI:** 10.2196/resprot.2725

**Published:** 2014-01-10

**Authors:** Konstantinos Antypas, Silje C Wangberg

**Affiliations:** ^1^Norwegian Center for Integrated Care and TelemedicineUniversity Hospital of North NorwayTromsøNorway; ^2^Department of Clinical MedicineFaculty of Health SciencesUiT The Arctic University of NorwayTromsøNorway; ^3^Narvik University CollegeNarvikNorway; ^4^Regional Center on Substance UseUniversity Hospital of North NorwayNarvikNorway

**Keywords:** focus group, design methodology, user involvement, user needs, health behavior models, tailoring, SMS, Internet, cardiac rehabilitation, smoking cessation, physical activity

## Abstract

**Background:**

Internet-based physical activity interventions have great potential in supporting patients in cardiac rehabilitation. Health behavior change theories and user input are identified as important contributors in the effectiveness of the interventions, but they are rarely combined in a systematic way in the design of the interventions.

**Objective:**

The aim of this study is to identify the appropriate theoretical framework, along with the needs of the users of a physical activity intervention for cardiac rehabilitation, and to combine them into an effective Internet- and mobile-based intervention.

**Methods:**

We explain the theoretical framework of the intervention in a narrative overview of the existing health behavior change literature as it applies to physical activity. We also conducted a focus group with 11 participants of a cardiac rehabilitation program and used thematic analysis to identify and analyze patterns of meaning in the transcribed data.

**Results:**

We chose stage-based approaches, specifically the transtheoretical model and the health action process approach as our main framework for tailoring, supplemented with other theoretical concepts such as regulatory focus within the appropriate stages. From the thematic analysis of the focus group data, we identified seven themes: (1) social, (2) motivation, (3) integration into everyday life, (4) information, (5) planning, (6) monitoring and feedback, and (7) concerns and potential problems. The final design of the intervention was based on both the theoretical review and the user input, and it is explained in detail.

**Conclusions:**

We applied a combination of health behavioral theory and user input in designing our intervention. We think this is a promising design approach with the potential to combine the high efficacy of theory-based interventions with the higher perceived usefulness of interventions designed according to user input.

**Trial Registration:**

Clinicaltrials.gov NCT01223170; http://clinicaltrials.gov/show/NCT01223170 (Archived by WebCite at http://www.webcitation.org/6M5FqT9Q2).

## Introduction

### Burden of Cardiovascular Diseases

The contribution of noncommunicable diseases to the burden of disease has increased over the last decades, especially in Western Europe. Cardiovascular diseases clearly have an important impact in this, ranked at the top of the causes of death with an increasing share in the burden of disease from 1990 to 2010 [1].

### Internet-Based Interventions to Support Physical Activity

In the same period the world experienced the explosive development of the Internet. Nowadays, use of the Internet is so widespread in many countries that it has become a popular means of delivering interventions to assist in diagnosis, treatment, prevention of illness, and the promotion of health. The number of health-related websites was estimated in the year 2000 to be more than 100,000, while today there are so many that it is not even possible to find an accurate estimate [2]. It would also be risky to estimate the general impact of Internet use on the burden of disease, but research shows that under certain conditions it can be a very useful tool in supporting self-management [3-9]. More specifically, there is the potential to influence physical activity that is very important for the prevention and the rehabilitation of cardiovascular diseases [7,10].

The effectiveness of Internet-based health interventions is connected with the adoption of the appropriate theoretical framework [11-14], while the viability of these interventions is associated with strong user involvement in their design [15]. For that reason, we are using a methodological approach that is combining the user-input and health behavioral theory to develop an Internet- and mobile-based physical activity intervention for cardiac rehabilitation.

Following our suggested process, we first review relevant models of health behavior and discuss our choice of the theoretical background for the intervention. We next present results from a user needs focus group, and finally, we describe the resulting design of the intervention.

## Methods

### Construction of Theoretical Framework

The choice of a theoretical framework is explained through a narrative overview and discussion of models of health behaviors in light of applicability to longitudinal tailoring. Then, theoretical concepts fitting well within different stages of behavioral change are reviewed.

### User Needs Focus Group

The user needs focus group took place in February 2010 at the Skibotn Rehabilitation Center in Norway. There were 3 women with mean age 64.3 years and 8 men with mean age 59.4 years, all attending the center’s cardiac rehabilitation program that month. The focus group was conducted at the center during the fourth and last week of the program’s duration and lasted one hour.

The discussion was based on an interview guide, but it was stressed that the goal would be an open discussion. The first part of the focus group was about needs, thoughts, and ideas of the users regarding support to increase physical activity and the corresponding role of technology. During this first part, the interviewers didn’t present any of the ideas for the intervention. The second part started with a very short presentation of some of the researchers’ ideas regarding the proposed intervention. The discussion that started in parallel with the presentation, and continued afterwards, focused on the opinions and reflections of the participants on the proposed concepts and intervention features. There were two interviewers that led the discussion.

The focus group discussion was audio recorded, verbatim transcribed, and analyzed with thematic analysis. Of the two researchers that analyzed the data, the first is a nurse with work experience in cardiovascular diseases. By the time of the focus group, the cardiovascular nurse had already developed some ideas regarding the intervention based on discussions with personnel at the rehabilitation center and on previous experiences with heart patients. The second researcher is a health psychologist with previous experience in developing Internet-based as well as tailored, health behavior change interventions, thus interpreting the data through glasses tinted by health behavior theories.

## Results

### Narrative Overview of Background for Theoretical Framework

#### Tailoring and Models of Health Behavioral Change

In this section, we present the rationale behind the choice of the theoretical framework for the Internet- and mobile-based intervention for physical activity. At first we explain why we use tailoring, an effective element of persuasive technology [15]. Then we present the different models of health behaviors, and how we combined them to comprise the core of the tailoring algorithm.

#### Tailoring

Bibliographic evidence is pointing toward the effectiveness and usefulness of tailoring. For example, perceived program relevance and amount of the materials read are found to be mediators of the effect of an Internet-based smoking cessation program [16]. A tailored intervention is one that is adapted to the characteristics of the individual, typically based on responses to a questionnaire [17]. Tailoring relies on three main methods: (1) personalization, (2) adaptation, and (3) feedback [18]. Personalization involves referring to the recipient in the text on the basis of details such as first name, age, gender, or hometown. Adaptation concerns the content of the text itself, which can be tailored according to a variety of theories. Feedback is a widely used feature in which the recipient is informed about scores on a scale, and how to interpret the results. In newer, more complex tailoring, these features are often combined, and the components of the Internet-based intervention may also be tailored.

Tailored health messages are in general perceived as more interesting and personally relevant, liked better, read more thoroughly, discussed more, and remembered better compared to nontailored educational material [19-22]. Personalization shows the most consistent effects of being tailored to [23,24] and involves referring to the recipient in the text on the basis of details such as first name, age, gender, or hometown. This is consistent with self-referent encoding, that all information that we associate with ourselves, is more easily noticed, stored, and retrieved [25].

#### Models of Health Behavioral Change

##### Continuum Versus Stage-Based Models of Behavioral Change

Models for health behavior can be roughly divided into two categories: (1) continuum models, and (2) stage-based models [26]. Velicer and Prochaska [27] argue that Schwarzer’s [26] division between continuum versus stage-based models can be conceptualized as theories of behavior versus theories of behavior change. The continuum models, to a great extent, are based on correlational studies of predictors of an on-going behavior, whereas the latter, to a greater extent, have studied predictors of transitional processes into a greater readiness for change. For the purpose of clarifying different implications for tailoring, we will continue our discussion with Schwarzer’s dichotomy.

Continuum models describe antecedents of behavioral change with the implicit assumptions that the sum of these antecedents needs to be above a certain threshold for a behavior to occur. Models vary as to whether and which variables are necessary and sufficient for behavioral change to happen. For instance, several models agree that having an intention to perform a behavior is necessary (but not sufficient) for the actual behavior to occur. Stage-based models, on the other hand, assume that there are distinct stages characterized by specific cognitive processes and motivational needs that the individual should pass through in sequence to get to behavioral change.

##### The Intention-Behavior Gap

Researchers within both kinds of models agree that there is a “gap” between intention and behavior [28], but a discussion with important implications for interventions is whether (for instance) intention is a static (indicator) or a dynamic (and changeable) variable [27]. Before and after forming an intention is a common chasm across several stage-based models [26], and is also seen as an important distinction demanding different strategies in nontheoretical methods such as motivational interviewing [29], which has been successful in supporting people in changing a host of health behaviors [30,31], including those relevant to cardiovascular disease risk [32-35]. Tailoring based on the continuum kind of models would imply that one determines which variables are “low” and then aims the intervention at increasing these, while tailoring based on stage-based models will identify the stages and deliver an intervention directed at the described processes within the particular stage.

Noar et al [36] found in their meta-analysis of tailored interventions that those based on the Transtheoretical model (TTM) [37] had the greatest effect. They further found that the number and type of theoretical concepts tailored on, including stage of change and processes of change, were associated with behavior change [36]. In general, physical activity interventions based on the TTM have not been very effective. Adams and White [38] point out potential reasons why this may be-that physical activity is complex, and that several of the reviewed interventions might not have optimally operationalized the TTM concepts. In other words, how we tailor to the relevant needs and processes within each stage is at least as important as the overarching framework (ie, the stages).

##### Starting From Stage of Change

As the first step in our tailoring, the participants’ stage of change is assessed using the University of Rhode Island Change Assessment - Exercise 2 (URICA-E2) [39]. In the next step, they follow different paths depending on the stage, starting with feedback on the current stage. As can be seen in Table 1, in addition to the variables described in the TTM [37], we have added some specific constructs from other theories according to what we see to be a good fit to the relevant processes in each stage. These are described in more detail below, along with our operationalization of these constructs in the tailoring of our physical activity intervention.

**Table 1:** The five TTM stages enriched with well fitting constructs from several theories.

	Stages from the TTM				
	Precontemplation	Contemplation	Preparation	Action	Maintenance
Relevant psychological constructs in different stages	Consciousness raising [37], Regulatory focus [40,41], Values [29], Environmental reevaluation [37], Outcome expectancies [42], and Supporting autonomy [29]	Decisional balance [37] and Self-reevaluation [37]	Action planning [26], Coping planning [26], Implementation intentions [43], and Self-efficacy for action [26]	Contingency monitoring [37], Counterconditi-oning [37], Stimulus control [37], Helping relationships (TTM) [37], Social support, Self-monitoring rewards, and Self-efficacy for maintenance [26]	Self-efficacy for recovery [26] and Relapse prevention

##### Regulatory Focus

A variable that we tailor on when we deliver health information to those who are concerned with the pros and cons of behavior change (ie, those in the first two stages) is the individuals’ promotion- or prevention-goal orientation (regulatory focus). Regulatory focus theory [40,41] separates those who are primarily motivated by achievement and gaining rewards (promotion) from those who are more concerned about avoiding loss and risk of such (prevention). This has implications for the kind of health information the individual is most affected by, and consequently, how we frame health information. Latimer et al showed that tailoring to regulatory focus (ie, matching it to the individuals’ regulatory focus) could increase both physical activity [44] as well as fruit and vegetable intake [45]. Our participants are presented with a regulatory focus assessment (Regulatory Focus Questionnaire-RFQ)[41]. Depending on classification, the participants are sent either prevention- or promotion-framed SMS text messaging (short message service, SMS) messages concerning physical activity over the next two weeks.

##### Decisional Balance

The balance between the pros and cons of behavior change has been shown to predict readiness to change across a host of health behaviors [46]. Our contemplators are presented with a decisional balance questionnaire [47]. The participant is then presented with immediate feedback according to whether they perceive more pros or cons with regard to regular physical activity. Next, the participant is presented with a list of potential reasons for becoming more physically active, and asked to tick off the relevant ones, before being asked to add some more in free text. This list is displayed on “My Page.” “My Page” is the profile page of the intervention where the most important information, the activities and the interaction of the user, and of their friends, are presented as a feed. A more detailed description of the functionality, as developed in combination with the user input, can be found in the section “Functionality” of the website.

##### Planning

In the planning phase of another stage-based model, the Health Action Process Approach (HAPA) [26,48], one separates action planning from coping planning [49,50]. Action planning refers to the planning of where, when, and how to perform the target behavior, and is thus similar to Gollwitzer’s concept implementation intentions [43]. Coping planning, on the other hand, concerns strategies for dealing with anticipated barriers, and is thus strongly connected to self-efficacy. From the preparation stage and onwards, the participant is asked to plan their physical activity in the “Exercise Agenda.” There, they can add several entries by planning what kind of activity, when, and where for each entry, thus forming an implementation intention. After completing planning, they are assessed for self-efficacy for this action plan. If it is very low, the user is asked to revise the action plan to make it more realistic.

##### Self-Efficacy

The concept self-efficacy refers to the degree to which an individual perceives that he or she can perform a particular behavior. The concept of self-efficacy stems from the social cognitive theory [42], but since self-efficacy is so closely related to behavior change, several researchers have assimilated it into other theories [26,29,51,52]. In the context of the two-stage HAPA [26], self-efficacy is important throughout behavior initiation, action, and maintenance, but HAPA distinguishes three kinds of self-efficacies: (1) action self-efficacy, you can perform the target behavior; (2) maintenance self-efficacy, you can maintain the target behavior despite barriers; and (3) recovery self-efficacy, you believe that you can succeed after a setback. While action self-efficacy in the HAPA model is directly related to intention, it is only indirectly related to behavior mediated via intention. Maintenance and recovery self-efficacy are on the other hand not related to intention, but directly related to behavior [26]. All these self-efficacies are assessed in the preceding stage. If the self-efficacy is low or moderate, the participant receives SMS messages concerning self-efficacy for the relevant stage over the next two weeks and also is asked to identify potential barriers (selecting from a list and in free text), and to generate strategies to address them. Strategies are then listed on “My Page.”

##### Social Support

Social support is important both directly for health status and via its influence on health behaviors [53]. In the TTM, social support is referred to as helping relationships and is seen as relevant to the action stage [37]. Social support is also found to increase throughout the stages [54]. We assess and give immediate feedback on social support in the preparation stage.

##### Relapse Prevention

Relapse prevention is trying to identify, prevent, or prepare to deal with high-risk situations. The most important goal is to make a plan for getting back to the plan; to prepare for continuing with the new health behavior in the event of a lapse, rather than giving in, perceiving the situation as all gains are lost, and all effort wasted, thus turning the lapse into a full-blown relapse [55]. Relapse prevention is mostly considered in relation to giving up substance use (eg, smoking cessation) [56], but we consider it relevant for other health behaviors too, and send SMS messages about relapse prevention to those of our participants in the maintenance stage that have indicated low to moderate self-efficacy for maintenance.

### The Focus Group

#### The Seven Themes

**Figure 1:**
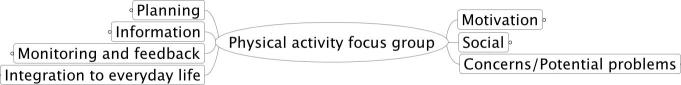
Thematic map of the focus group themes.

There are seven main themes that were identified in the focus group: (1) *social*, (2) *motivation*, (3) *integration to everyday life*, (4) *information*, (5) *planning*, (6) *monitoring and feedback*, and (7) *concerns/potential problems*. The themes are presented in the thematic map (Figure 1 shows these themes, also see Multimedia Appendix 1 for these themes). The results and the thematic map presented in this paper are a slightly more revised version than the one used for the development of the intervention, in the direction of improved synthesis of the data.

#### Social

The largest pattern of meaning that appeared in the focus group was the *social* theme. In addition to its high level of frequency, this theme is the one that included the most subthemes and codes. Under this theme we have included ideas, thoughts, and needs, expressed by the users referring to companionship, belonging to a group, or communication with others.

One of the dimensions of this theme repeatedly expressed by the participants was the importance of not being alone in the behavior change endeavour. This was an important factor that helped them during their stay in the rehabilitation center, and it was something that they wished to maintain after they were discharged. In some cases, they were referring to the importance of staying connected with the very same people with whom they shared the rehabilitation program.

I am like this, that I need a bit of this motivation from the others also, to try alone, this is…This is the problem…so...this here with the local team, this can be a reasonable angle of this also, or approach eeh…attach yourself to the local team, also continue this you have started with them now…for example.

Male

The importance and the benefits of belonging to a group were further explained. Peer support is the main benefit the participants seemed to enjoy at the rehabilitation center, and is one of the mechanisms through which they can help each other.

A forum of course is also something to talk about, a brilliant thing…talk with each other in a forum and ask things…put out eeh…

Male 1

you should have a forum only between… peers…

Male 2

As expected, the peer support appears to be connected with the functionality of the forum. In the next extract, we also see the concept of the social obligation that the participants recognize as a possible mechanism to maintain or increase physical activity. The participants feel the obligation to do something that their peers are doing or ask them to do.

I am saying that if we have it fixed, one time per week, that we send a message to each other and then, then you feel committed to say yes, for as long as you like…Yes, then you must have something else that really, you have something else that you have to do, or else…you just do it.

Female

In another instance, the social obligation is connected with a request for a training diary combined with the forum.

Training diary on the Internet…And also have a group where someone can subscribe to a forum, or have a…to brag…yesterday I walked for an hour and today I have been to the training…and tomorrow I have thought, yes…So, it is like this that someone gets to, a bit, a bit like a competition, internally between each of us. We will train, as much as possible we will commit to ourselves a bit more also.

Male

Commitment is also related to a healthy competition with each other. Through the forum, the participants suggested that they would succeed simply to encourage each other, an important mechanism related to the *social* theme.

Yes, yes I think that for many…if you take as basis the situation we are in now and you want to prolong it as long as possible, all of us want to stay here four weeks more, isn’t it? And four weeks after that, life is great here…But to stay in touch with the “gang,” so, so I think that the most of us would think that, yes, the Internet, the approach that is best, I don’t have any faith in SMS, but eeh, Internet, a forum yes. To keep up, keep the spirit of the team up, the mood, the good flow.

Male

The participants also had specific suggestions regarding the functionality of the forum. For example, they were positive about having two levels of access, one reserved only for the members of the same monthly group. In this level, they would like to share photos with the other members, maintaining their bonds after the rehabilitation stay. The social dimension of the forum was not only mentioned in relation to the other participants, but also included the personnel of the center. The participants mentioned that they would like to know that at the forum there are health professionals they can trust to answer their questions.

Yes, there should be someone that can answer, that has a clue and that can answer.

Male

There should be professionals too…yes.

Female

The fact that Facebook is the largest and most popular social network, and one of the most popular websites in Norway, can explain that users were often inspired by Facebook functionality, and sometimes even explained a desired functionality as “like Facebook.” In the same context, the concept of a training buddy was also popular. That is a person that would be paired with that participant, and they would support each other possibly with their physical presence, but mainly through the interaction the Internet tool would provide.

Almost like Facebook that…A forum is a living thing, like you come here and just are…new things pop up all the time and…between users…it’s alive. (…) Do you want to be my workout friend? (Laughter from the rest of the group)

Male

Regarding the choice of technology that would support the social functions of the intervention, participants mentioned the Internet and SMS in both parts of the focus group (in the general discussion and the discussion after the initial ideas were presented). One participant was sceptical to the usefulness of the SMS, but this didn’t reflect the opinion of the rest of the group.

#### Motivation

The theme *motivation* includes ideas and methods believed to influence or capable of influencing the participants’ behavior. The theme includes the strong belief that the responsibility for change of behavior is personal. The participants mentioned it mainly in relation to what is going to happen after they leave the rehabilitation center.

I believe actually, I believe that someone gets used to it, if we make a system, habits. That it doesn’t get too much, that we know that…we go online…and we get our own responsibility of our own training.

Male 1

It is not, it is not that anyone says that you have to cycle. Also, it is made that each does what himself/herself feels.

Male 2

So, so, it requires self-discipline.

Male 3

Making a decision to prioritize themselves and the behavioral change was also very central. Prioritization was discussed in several instances as a method to maintain physical activity and generally continue the changes in behavior after the discharge.

That we chose to prioritize the demands others have of us.

Female

Down-prioritize ourselves all the time.

Male 1

Got to be better at saying–“No thanks, today I can’t.” But on Tuesday it doesn’t work either, for I’m exercising”

Male 2

#### Integration to Everyday Life

Another theme that emerged from the focus group was *integration to everyday life*. The participants often referred to thoughts and things to do that are helping them to integrate a desirable behavior into their everyday life. In the same way, the participants wanted technology that would assist them in maintaining the desired behavior in a way that also integrates it into their everyday life. Special emphasis was placed on simple changes in the activities of their everyday life that can increase physical activity.

I think that someone should not have high expectations of himself, that would make him strive to get there. I believe that you get tired of it, I think you should have only simple changes in your life.

Male

The participants also discussed that creating new habits is helpful in changing behavior by integrating the desired behavior into everyday life, mainly by replacing old bad habits. The reverse order also seemed to be possible. By integrating an activity or even a technology into everyday life, an old habit could be replaced with a new good one that would assist in changing behavior.

I believe in small simple things like in everyday life, that if someone manages to walk to the store or walk to work maybe…things like that can also be important, instead of taking the elevator, if you are working in a building that you can take the stairs instead of the elevator, if you do it often, it is not bad either…instead of sit in the car and drive a few meters, to walk to the shop instead, so can someone ride a bicycle when it is becomes summer, or go with the chair-sledge…that someone can do things like that, it gets possible. Someone becomes so lazy that doesn’t bother, sits in the car, the old habit, instead of just walking.

Male

A technology that would help to integrate the desired behavior into everyday life should also be integrated into everyday life. Ubiquitous technology can support behavior change in the challenging situations of everyday life, or remind users of their own commitments.

If you could get a message every day, there and then?

Male 1

Have you been good today? No, now you have to go out, time for exercise.

Male 2

Get out you lazy bastard!

Male 1

And it should come on a specific time you have decided to walk today, or go out…

Male 2

Or even better, a couple of hours before…so you won’t change your mind.

Male 1

#### Information

Despite being in a rehabilitation program where they could have access to all the information they needed, or maybe because they were there and were experiencing good access to information, the participants of the focus group expressed their need for tools that would help them access the right information for a long time after their discharge. They referred repeatedly to the need to find the right answers, either through a forum or a kind of knowledge bank. They also wanted the health professionals to take an active part in the forum, and specifically for physical activity, provide suggestions for training plans.

It should be a forum where you have the opportunity to get…eeh…get the right answers, […] access to a resource, this is what I believe it becomes. It has an effect.

Male 1

What is good with a forum is that everything that is asked and discussed and answered…it stays there, you don’t even have to ask, if…if it [the forum] is used a lot, you can just with a simple search find what you need…The hope is that it will become a kind of knowledge bank. And the problems you have experienced like pain and things that you can go in and have a look and talks to others about them.

Male 2

#### Planning

This theme covers a very effective part of the behavior change process. The participants expressed the necessity to plan in advance their physical activity in order to actually do it. First of all, the plans have to be realistic in order to make it possible to achieve them.

[…] if we were sportsmen, we would have to climb extremely high. As you say, leave the car, to walk a bit, we have made a lot.

Male

Maybe it is a bit of [your] responsibility, a time schedule with realistic goals.

Female

The technical dimension of this theme was expressed with a clear request for a training diary that would help the users plan activity, preferably on a weekly basis so they would avoid being drawn to their old way of behaving, where physical activity was constantly neglected.

[…] I believe that what is important with the schedule is that you set it off, you prioritize a bit, you see that okay, that and that day it passes better maybe. So you say at those two days or three days a week, they are mine, then I should train. If you don’t make it to a system, it gets difficult, easy to neglect, if you don’t put aside time for it, because then it is so many other things that comes in front all the time. Then it becomes neglected, this is anyway my experience. But if you, like what I did before Christmas, before I came here, then I decided that in the evenings I should be going on walking tours. When the children go to lie down, I am going for a tour. And I did it. […] It should come first…or we die, this is how I am thinking…

Male

#### Monitoring and Feedback

The theme of *monitoring and feedback* appears in the second part of the focus group, during the discussion and after the presentation of some specific ideas for the intervention. It refers to the necessity and the requirements related to keeping a record of the physical activity of the users, and how to present it in a meaningful way to them. The discussion was dominated by the previous experiences of some of the participants with monitoring sensors, feedback statistics, and graphs, mainly from commercial products and services. The rest of the group was also interested even if they had no personal experience with the sensors, and generally were positive to the idea.

After what I have seen, there is a whole program, and shows graphically also how the climb has been, also the mountains, everything is there. There is also the pulse there. I have been many times in on the Internet and seen how the whole training of the day has been. […] And there you can see them, there is graphic representation, how it has been, up the hill, down the hill and…

Male

#### Concerns and Potential Problems

Despite the positive reception to the idea of an intervention, several concerns and fears for potential problems were expressed during the focus group. Regarding the process of changing behavior, the participants of the focus group were concerned about the obstacles they have to overcome in their effort to maintain or increase physical activity. Lack of training facilities, lack of time, or just going back to their everyday life are possible obstacles that make them question their ability to maintain the desired behavior.

We have developed some habits while here. This, this I believe we cannot manage outside. And then we maybe cannot maintain, keep enough habits. We haven’t…[…] I live far from people and fatherland, to say it like this. […] Yes, my place is far [from a city]…we have no swimming pool or any big activities. We should just organize our own activities…when we live far, far in a village.

Male 1

For example, 52 weeks that I can use them as I want, but I should try to use them right. [Me] and someone else that has to go to work, we cannot do it equally.

Male 2

Along with the concerns about the obstacles in changing behavior were the concerns about relapsing from the desired behavior. For various reasons individuals might stop being physically active for short or long periods of time, and for that case, the participants expressed a need for support to get them back on track.

And when you come home and you get back pain, you don’t manage to keep up, so you become disappointed and sad, and you feel that, no, I am a loser.

Male

The major concerns though, were expressed for the technology. When it came to the usability of the website, the participants suggested that we should consider e-literacy issues and offer training to the users. They also recommended that the website be maintained in such a way that it is constantly updated, with the content remaining politically and religiously neutral and independent. A few participants were sceptical of the SMS technology, believing it was not answering the needs for behavior change. This view was balanced by the request of many other participants to send them reminders and motivational messages to trigger behavior change. The potential risk of high dropout rate from the forum was also discussed.

As in many Internet-based interventions, participants shared another important concern—privacy. They asked to have the choice on what to share with whom.

You can choose, if you want it…to make it public…Make it accessible for the others, so…it should be a keystroke or a choice you do […] In periods it might be like that, that you don’t want to show it…

Male

A female participant also expressed a concern regarding the ability of an intervention to cover the needs of women too. This shows the need for a gender- sensitive approach, mainly by offering additional training where it is needed. Finally, tailoring, a concept very central to the suggested intervention caused scepticism in a few participants. They questioned the ability of technology to provide a satisfying degree of personalization.

But it is not possible that you [have] many different [categories], because someone is not so individual that cannot fit in maybe four different…I am thinking like this…I want to believe that to make conclusions from the questionnaire there, that has maybe four different categories…do you know what I mean?…I answer in this way, I belong to category four, Ola answered that, he belongs in category three, in this way, a bit slack it is,…If it is tailored to 12 different [categories], then it becomes, it becomes very ambitious, I think…

Male

### The Design of the Intervention

The researchers translated the combination of the theoretical framework and the user input into the technical requirements documentation that was later used by an external collaborator to develop the actual intervention. In Table 2, we list the contribution of the theoretical constructs and the relevant focus group themes in each functionality element of the intervention. The intervention is based on the popular open source content management framework Drupal. The main phase development that produced a functional prototype lasted one year. It was followed by a second phase of six months that included bug fixing by the external collaborator, the implementation of the tailoring algorithm into Drupal, testing, and content development by personnel of the Norwegian Center of Integrated Care and Telemedicine and the Skibotn Rehabilitation Center.

**Table 2:** Contribution of theory and user input in the functionality included in each element of the intervention.

Elements of the intervention	Relevant theoretical constructs	Relevant focus group themes
Levels of access	-	*Social* and *concerns/potential problems*
Microblog functionality of “My Page”	Social support, Consciousness raising, and Helping relationships (TTM)	*Social* and *motivation*
Activity calendar	Preparation (TTM), Action planning, Contingency monitoring, and Self-monitoring	*Planning, monitoring and feedback, social, information, motivation*, and *integration to everyday life*
Discussion forum	Social support and Helping relationships (TTM)	*Social* and *information*
General information	Consciousness raising and Self-efficacy for action	*Information*
Contact with physiotherapist and technical support	Social support	*Social, information,* and *concerns/potential problems*
Weekly activity goal on “My Page”	Outcome expectancies, Self-efficacy for action, Action planning, Implementation intentions, and Supporting autonomy	*Motivation, planning,* and *monitoring and feedback*
Simple feedback graph on “My Page”	Outcome expectancies, Self-reevaluation, and Consciousness raising	*Monitoring and feedback, information*, and *planning*
My reasons for physical activity, my strategies to increase physical activity, and overcome barriers on “My Page”	Self-efficacy for maintenance and recovery, Values, Coping planning, Relapse prevention, and Decisional balance	*Motivation, integration to everyday life, information,* and *concerns/potential problems*
Tailoring algorithm	Mainly based on theoretical constructs (see detailed explanation under Theoretical framework and Table 1)	*Motivation, planning, social, information, integration to everyday life,* and *concerns/potential problems*

### Functionality of the Website

#### The Graphic Design

The graphical design of the website was based on the graphic profile of the patient organization that owns the collaborating rehabilitation center. Since the offer of the intervention is an extension of the services offered by the face-to-face rehabilitation, it is important to use the visual elements and a palette familiar to the users and identical to the website of the rehabilitation center, to the website of the patient organization that owns the center, and to all the printed materials that are used by the organization and all its services. Since the patient organization is one of the biggest and most active in Norway, we expect that the level of trust toward our intervention will be positively affected. The main colors of the website are blue and light blue, which, according to the graphic profile of the organization, have been chosen because they symbolize clean air and breath. For the typography, modern, but simple fonts have been selected to make the information easy to read.

#### Three Levels of Access

There are three levels of access for most of the components of the website. The first one is information accessible by all the registered users of the website. The second level is accessible only by a specific group of users that are called friends. Friends are by default the participants of the same rehabilitation monthly group, for example, participants that have been to the rehabilitation center in January 2012. A user can add or delete friends from her profile page. A third level of access is reserved for the user and the information that is private. Users with administrative roles can have access to information of all levels.

#### My Page

**Figure 2:**
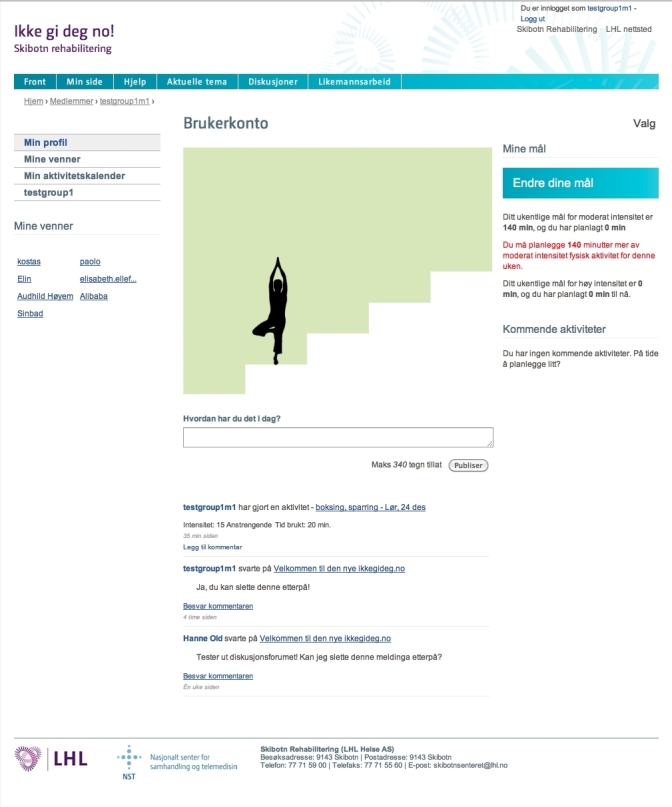
The profile page (My Page).

For the user, the starting point is the profile page, called “My Page” (Figure 2 shows this page). The profile page includes a wall functionality that could also be described as a microblog functionality, where the planned and completed activities from the calendar appear. The user can write how they feel in general or about the activities they have planned, and can see other users’ posts. To avoid lengthy posts, a limit of 340 characters is applied. For each post on the profile, there is the possibility for the friends of the user or the center’s personnel to comment. From the profile page, the user can access the friends’ list and the personal information page. The most recently planned physical activities appear also on the side of the profile page. There is also a link to the group page. The group page is similar to the profile page, but only shows the latest activities of the members of the monthly group to which the user belongs.

#### Activity Calendar

**Figure 3:**
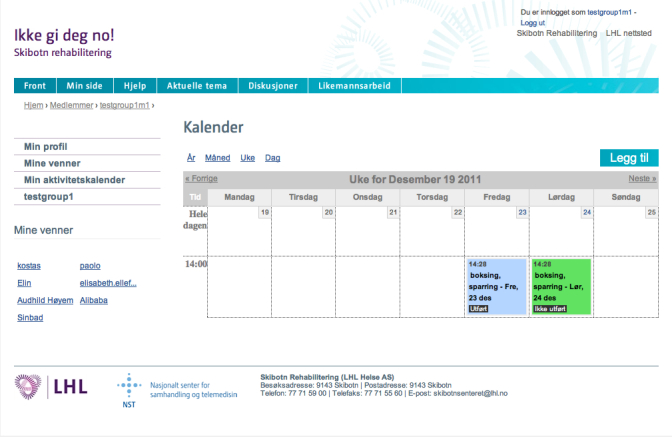
Weekly overview of the planned activities of the activity calendar.

**Figure 4:**
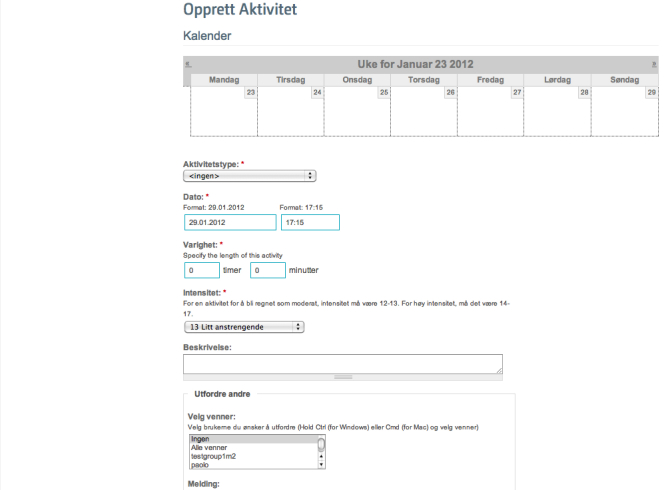
Planning of an activity in the activity calendar.

**Figure 5:**
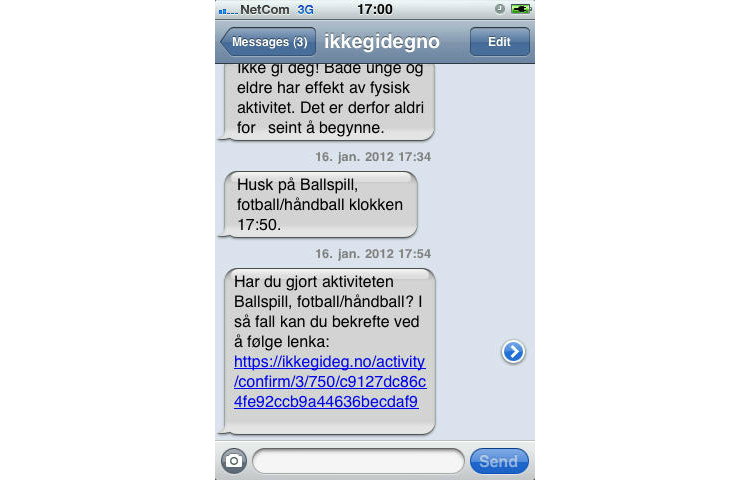
SMS before and after planned activity: "Remember Ball game, football/handball at 17:50 (second SMS from below)," "Did you do the activity Ball game, football/handball?" "If so, you can confirm it by following the link [unique URL]" (first SMS from below).

**Figure 6:**
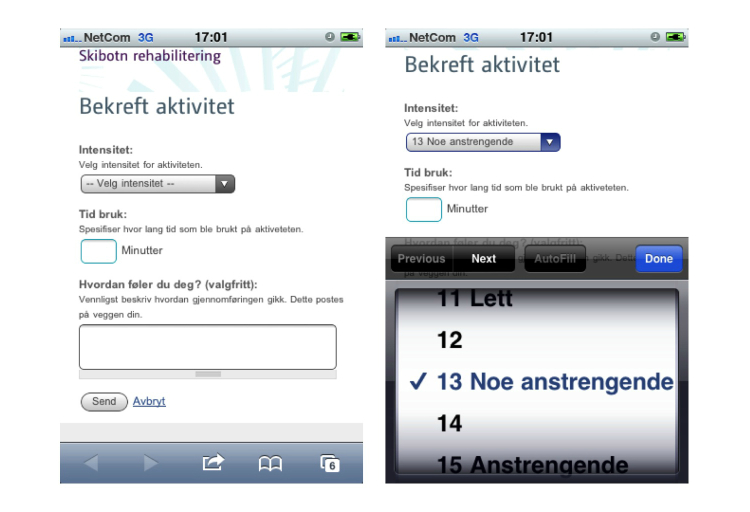
Mobile page for confirming the completed activity. Questions about the intensity of the activity, actual duration of the activity, and free text comment field (left image). The intensity of the activity according to Borg’s scale (right image).

The activity calendar is a planning and reminder tool. The main view is the weekly calendar (Figure 3 shows this weekly view), since the users are encouraged to plan activities on a weekly basis, but daily, monthly, and yearly views are also available. The completed activities appear on the calendar with a different color, and the user can edit both the completed and planned activities. To plan an activity, the user can choose from a preselected list with common activities (Figure 4 shows this page). The user has to set the start date and time, duration in hours and minutes, and planned intensity according to Borg’s scale [57]. In addition, the user can choose to make the activity public or private, to write the place of the activity, and to provide an additional description. An important functionality is that there is the possibility to challenge some or all of their friends to take part in the activity by inviting them through the same page. The invited friends will see an invitation on their profile page and will also get an email and an SMS invitation. For each planned activity, the user will receive an SMS reminder 15 minutes before the planned beginning of the activity, and an SMS at the planned end asking about the completion of the activity (Figure 5 shows this reminder). The last SMS contains a URL link that users with smartphones can use to confirm their activity as completed and state the actual duration of the activity, the actual intensity, and write a comment if they want (Figure 6 show this page). Users without a smartphone can update the information the next time they log in on the website. The information about the completed activity is published on the profile of the user and can also be seen by their friends on their page.

#### Discussion Forum

**Figure 7:**
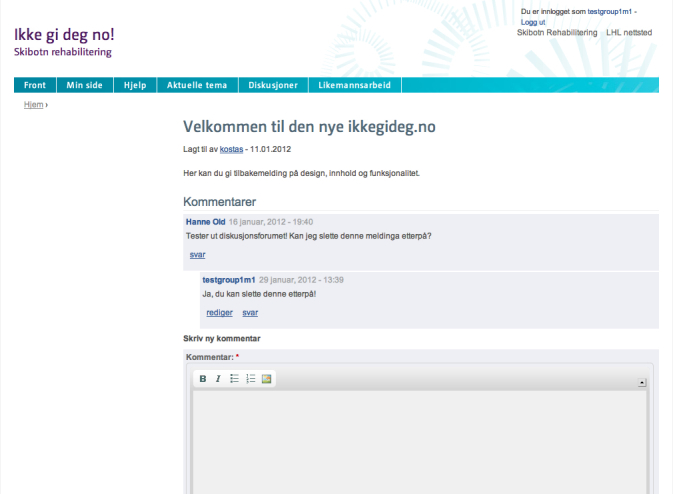
The discussion forum.

The discussion forum is a standard discussion forum with three levels of access (Figure 7 shows the forum). The first one is only accessible to all the registered users of the website. The second level is for discussions that are only accessible by users that belong to the same monthly group. The third level of access is reserved for the administrators and the health professionals that can access all the discussions to moderate and give professional advice and motivation.

#### General Information

The health professionals involved in the project have developed and posted on the site general information regarding physical activity and training, cardiovascular disease, diabetes, lung problems, cholesterol, smoking cessation, and other topics that are relevant for the users. There is also information regarding motivation, self-management, and lifestyle change that are closely related to the concepts the intervention is built upon. The information is accessible to all the visitors of the website, reflecting requests from participants of the rehabilitation program. It is a reliable and verified resource.

The users can seek assistance in navigating the website by calling a physiotherapist responsible for it, during working hours. For technical issues, they can complete an Internet-based form and submit their comment or problem. When possible, they receive a response within three working days.

#### Additional Functionality for Intervention Group

Some additional functionality is available only for the members of the intervention group of the randomized controlled trial [58]. On the profile page, those users can see their weekly activity goals. The users set the weekly goals on a new page as minutes of activity in two categories: (1) high intensity, and (2) moderate intensity. The users receive feedback regarding the level of activity in comparison to the suggestions by the American College of Sports Medicine that are much in line with the suggestions from the Norwegian Directorate of Health [59]. The feedback is offered as advice and the user can proceed even without complying with the suggestions, this is to reflect the individual needs and exercise capacity of the user and the focus on self-management. The goals appear on the side of the profile page and are accompanied by feedback related to the planned activity and how it compares to the set weekly goals. Related to the achievement of the goals, is the graph that appears on the profile page. In a simple feedback graph, the user appears as a figure on a ladder with 5 steps and, according to completed activities, the figure is on one of the 5 steps (Figure 2). The figure on the top of the ladder represents the successful completion of the weekly goals, and it appears to be in a more cheerful position than when on the base of the ladder. The figure is different for male and female users. On the side of the profile the user can find certain strategies to increase physical activity or overcome barriers (depending on the stage of change of the user), and their most important reasons to be more physically active, again chosen by the user. The strategies and the reasons appear only for users in specific stages and the users have either chosen them from a list of suggestions or written them by themselves.

### Tailoring Algorithm

#### Stages of Change

As the first step in tailoring, the participants’ stage of change is assessed using the URICA-E2 [39]. In the next step, they follow different paths depending on stage.

#### Precontemplation

The participant is given immediate feedback on the current stage, and is then asked whether they would like to test their knowledge about physical activity. If yes, a quiz on benefits of physical activity is given, followed by the results. Then the participant is presented with a RFQ [41]. Depending on classification, the participant will be sent either prevention- or promotion-framed SMS messages concerning physical activity over the next two weeks (see Multimedia Appendix 2). After two weeks the participant is reassessed for stage of change.

#### Contemplation

The participant is given immediate feedback on the current stage, and is then presented with a decisional balance questionnaire [47]. Afterwards, the participant is presented with immediate feedback according to whether they perceive more pros or cons regarding regular physical activity. Next, the participant is presented with a list of potential reasons for becoming more physically active, and asked to tick off the relevant ones, before being asked to add some more in free text. This list is displayed on “My Page.” The participant is then asked if they want to plan their physical activity. If yes, they are presented with the “Exercise Agenda.” There, they can add several entries by planning what kind of activity, when, and where for each entry, thus forming an implementation intention. After completing planning, they will be assessed for self-efficacy for this action plan. If it is very low, the user will be asked to revise the action plan to make it more realistic, while if it is moderately low, they will receive SMS messages concerning self-efficacy for action over the next two weeks. If the participant declines planning, they will be led to “My Page.” All participants will be reassessed for stage again in two weeks.

#### Preparation

The participant is given immediate feedback on the current stage, and then self-efficacy for their action is assessed. The participant is asked to identify potential barriers and to generate strategies to address them. These strategies are then listed on “My Page.” Next, social support is assessed and immediate feedback is given. Then, the participant is asked to plan physical activity in the activity calendar. Over the next two weeks the participant will receive SMS messages about self-efficacy and/or social support, depending on the above assessment, before stage is reassessed (see Multimedia Appendix 2).

#### Action

The participant is given immediate feedback on the current stage, and then self-efficacy for maintenance is assessed. The participant is asked to identify potential barriers and to generate strategies to meet them. The strategies are then listed on “My Page.” The participant is also asked if they want to update their activity calendar. After planning activities, they are asked about self-efficacy for this plan. Over the next two weeks SMS messages about maintenance are sent to those who were low on self-efficacy for this, and then stage is reassessed.

#### Maintenance

The participant is given immediate feedback on the current stage, and then self-efficacy for relapse is assessed. The participant is asked to identify potential barriers and to generate strategies to meet them. The strategies are then listed on “My Page.” The participant is then asked if they want to update their activity calendar. After planning activities, they are asked about self-efficacy for this plan. Over the next month SMS messages about relapse prevention are sent to those who were low on self-efficacy for this, and then stage is reassessed.

## Discussion

### Communication Design

The communication design of a website is an essential component of the intervention. There are several factors that have to be considered in relation to the target group and the communication channel that is going to be used, such as font style and size, balanced use of graphics and text, and intuitive structure and navigation menus [60,61]. Building credibility also has great potential; since it seems that it affects the confidence in one’s thoughts, health behavior, and cognition [62]. This can be effectively done with visual and design cues [63], and for our intervention this was applied with the right choice of communication design elements like template, colors, fonts, and of course the logos of the organizations.

### Theoretical Implications

In this paper, we describe how we developed an intervention in which the existence of each of its functionality elements is grounded on both user input and theoretical constructs. Of course, the health behavior models that we used to create the theoretical framework were developed based on research of human behavior, which to a certain extent qualifies for user input. It was expected that those concepts about human behavior would be reproducible and would reappear in our focus group.

An example of the reflection of theory in the focus group appears in the themes of *planning* and *motivation*. According to the HAPA, there is a distinction between action planning and coping planning [49,50]. The participants of the focus group were clearly concerned in a different way about planning an activity, compared to preparing to cope with the barriers of physical activity once they completed their rehabilitation stay. It seemed that both are necessary, but prioritization and motivation are needed to make sure each participant will be ready to overcome any difficulties.

Another example of integration is concerning relapse prevention [64,65]. The focus group confirmed its relevance for the case of physical activity. The participants mentioned that after a health problem, like back pain, they might backslide and find it difficult to start physical activity again. For that reason, they would like to get support in dealing with such feelings as disappointment, sadness, and being a loser, in order to recover. Also, the relationship of the social support with the relapse prevention, as seen in the focus group, is coherent to the previous findings that indicated that social support is related to the resistance of relapse into physical inactivity in men [65].

Whereas the stage-based models that we applied may carry some merit for creating tailoring algorithms, they are not sufficient in accounting for all the determinants of physical activity [38]. Within health promotion, more ecological models [66] are used, including factors from within the individual, via the closest network, community to societal regulations, and resources. Although more inclusive, these kinds of models raise a number of methodological and logistical challenges [66]. Some, such as the purely Internet-based, might be even more difficult to tackle, while others might benefit from the improved trackability of an Internet-based intervention. A somewhat more limited ecological model that would fit well to our existing variables, while including more of the social ones, is the social cognitive theory [67]. Whereas self-efficacy is the most important variable in the social cognitive theory, social variables play several important roles-they influence our expectations about outcomes, self-efficacy, and they constitute direct facilitators as well as impediments for behavior. Thus, in a temporal perspective, social variables are important throughout the stages of behavioral change.

Nevertheless, as we add more variables to our models, we should be careful to measure our proposed mediators to make sure that we are actually intervening according to our proposed theoretical framework [68]. By gathering data on the relevant processes hypothesized to take place, not only can we further develop our interventions, but our theories as well [69]. We therefore aimed to design the randomized controlled trial of the current intervention [58] so that in addition to being able to conclude whether the intervention was effective or not, we will know something about what works and why. Ideally, we believe, the design of the intervention and the design of the trial should go hand in hand.

## Multimedia Appendix 1

Interactive thematic map of the focus group themes and subthemes.

## Multimedia Appendix 2

Video presenting an example of the tailoring algorithm.

## Abbreviations

HAPA: Health Action Process Approach
RFQ: Regulatory Focus Questionnaire
SMS: short message service
TTM: Transtheoretical model
URICA-E2: University of Rhode Island Change Assessment – Exercise 2
