# Supplementary material for: Combining Users’ Needs With Health Behavior Models in Designing an Internet- and Mobile-Based Intervention for Physical Activity in Cardiac Rehabilitation
Source: JMIR Res Protoc. 2014 Jan 10;3(1):e4. doi: 10.2196/resprot.2725 (PMC3913925; doi:10.2196/resprot.2725)
Supplement: Supplementary file 1 [file resprot_v3i1e4_app1.zip › Interactive thematic map/thematic map v5.html]

xml version="1.0" encoding="UTF-8"?
Physical activity Focus group 

Flash plugin or Javascript are turned off.
Activate both and reload to view the mindmap
