# Supplementary figures and images for: Combining Users’ Needs With Health Behavior Models in Designing an Internet- and Mobile-Based Intervention for Physical Activity in Cardiac Rehabilitation
Source: JMIR Res Protoc. 2014 Jan 10;3(1):e4. doi: 10.2196/resprot.2725 (PMC3913925; doi:10.2196/resprot.2725)

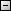

Supplement: Supplementary file 1 [file resprot_v3i1e4_app1.zip › Interactive thematic map/thematic map v5.html_files/hide.png]

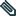

Supplement: Supplementary file 1 [file resprot_v3i1e4_app1.zip › Interactive thematic map/thematic map v5.html_files/icons/attach.png]

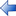

Supplement: Supplementary file 1 [file resprot_v3i1e4_app1.zip › Interactive thematic map/thematic map v5.html_files/icons/back.png]

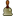

Supplement: Supplementary file 1 [file resprot_v3i1e4_app1.zip › Interactive thematic map/thematic map v5.html_files/icons/bell.png]

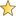

Supplement: Supplementary file 1 [file resprot_v3i1e4_app1.zip › Interactive thematic map/thematic map v5.html_files/icons/bookmark.png]

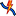

Supplement: Supplementary file 1 [file resprot_v3i1e4_app1.zip › Interactive thematic map/thematic map v5.html_files/icons/broken-line.png]

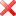

Supplement: Supplementary file 1 [file resprot_v3i1e4_app1.zip › Interactive thematic map/thematic map v5.html_files/icons/button_cancel.png]

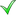

Supplement: Supplementary file 1 [file resprot_v3i1e4_app1.zip › Interactive thematic map/thematic map v5.html_files/icons/button_ok.png]

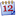

Supplement: Supplementary file 1 [file resprot_v3i1e4_app1.zip › Interactive thematic map/thematic map v5.html_files/icons/calendar.png]

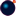

Supplement: Supplementary file 1 [file resprot_v3i1e4_app1.zip › Interactive thematic map/thematic map v5.html_files/icons/clanbomber.png]

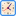

Supplement: Supplementary file 1 [file resprot_v3i1e4_app1.zip › Interactive thematic map/thematic map v5.html_files/icons/clock.png]

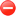

Supplement: Supplementary file 1 [file resprot_v3i1e4_app1.zip › Interactive thematic map/thematic map v5.html_files/icons/closed.png]

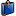

Supplement: Supplementary file 1 [file resprot_v3i1e4_app1.zip › Interactive thematic map/thematic map v5.html_files/icons/desktop_new.png]

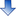

Supplement: Supplementary file 1 [file resprot_v3i1e4_app1.zip › Interactive thematic map/thematic map v5.html_files/icons/down.png]

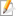

Supplement: Supplementary file 1 [file resprot_v3i1e4_app1.zip › Interactive thematic map/thematic map v5.html_files/icons/edit.png]

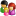

Supplement: Supplementary file 1 [file resprot_v3i1e4_app1.zip › Interactive thematic map/thematic map v5.html_files/icons/family.png]

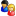

Supplement: Supplementary file 1 [file resprot_v3i1e4_app1.zip › Interactive thematic map/thematic map v5.html_files/icons/fema.png]

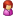

Supplement: Supplementary file 1 [file resprot_v3i1e4_app1.zip › Interactive thematic map/thematic map v5.html_files/icons/female1.png]

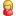

Supplement: Supplementary file 1 [file resprot_v3i1e4_app1.zip › Interactive thematic map/thematic map v5.html_files/icons/female2.png]

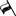

Supplement: Supplementary file 1 [file resprot_v3i1e4_app1.zip › Interactive thematic map/thematic map v5.html_files/icons/flag-black.png]

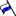

Supplement: Supplementary file 1 [file resprot_v3i1e4_app1.zip › Interactive thematic map/thematic map v5.html_files/icons/flag-blue.png]

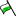

Supplement: Supplementary file 1 [file resprot_v3i1e4_app1.zip › Interactive thematic map/thematic map v5.html_files/icons/flag-green.png]

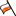

Supplement: Supplementary file 1 [file resprot_v3i1e4_app1.zip › Interactive thematic map/thematic map v5.html_files/icons/flag-orange.png]

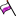

Supplement: Supplementary file 1 [file resprot_v3i1e4_app1.zip › Interactive thematic map/thematic map v5.html_files/icons/flag-pink.png]

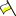

Supplement: Supplementary file 1 [file resprot_v3i1e4_app1.zip › Interactive thematic map/thematic map v5.html_files/icons/flag-yellow.png]

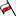

Supplement: Supplementary file 1 [file resprot_v3i1e4_app1.zip › Interactive thematic map/thematic map v5.html_files/icons/flag.png]

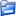

Supplement: Supplementary file 1 [file resprot_v3i1e4_app1.zip › Interactive thematic map/thematic map v5.html_files/icons/folder.png]

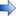

Supplement: Supplementary file 1 [file resprot_v3i1e4_app1.zip › Interactive thematic map/thematic map v5.html_files/icons/forward.png]

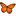

Supplement: Supplementary file 1 [file resprot_v3i1e4_app1.zip › Interactive thematic map/thematic map v5.html_files/icons/freemind_butterfly.png]

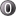

Supplement: Supplementary file 1 [file resprot_v3i1e4_app1.zip › Interactive thematic map/thematic map v5.html_files/icons/full-0.png]

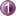

Supplement: Supplementary file 1 [file resprot_v3i1e4_app1.zip › Interactive thematic map/thematic map v5.html_files/icons/full-1.png]

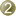

Supplement: Supplementary file 1 [file resprot_v3i1e4_app1.zip › Interactive thematic map/thematic map v5.html_files/icons/full-2.png]

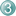

Supplement: Supplementary file 1 [file resprot_v3i1e4_app1.zip › Interactive thematic map/thematic map v5.html_files/icons/full-3.png]

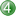

Supplement: Supplementary file 1 [file resprot_v3i1e4_app1.zip › Interactive thematic map/thematic map v5.html_files/icons/full-4.png]

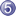

Supplement: Supplementary file 1 [file resprot_v3i1e4_app1.zip › Interactive thematic map/thematic map v5.html_files/icons/full-5.png]

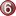

Supplement: Supplementary file 1 [file resprot_v3i1e4_app1.zip › Interactive thematic map/thematic map v5.html_files/icons/full-6.png]

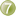

Supplement: Supplementary file 1 [file resprot_v3i1e4_app1.zip › Interactive thematic map/thematic map v5.html_files/icons/full-7.png]

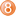

Supplement: Supplementary file 1 [file resprot_v3i1e4_app1.zip › Interactive thematic map/thematic map v5.html_files/icons/full-8.png]

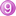

Supplement: Supplementary file 1 [file resprot_v3i1e4_app1.zip › Interactive thematic map/thematic map v5.html_files/icons/full-9.png]

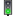

Supplement: Supplementary file 1 [file resprot_v3i1e4_app1.zip › Interactive thematic map/thematic map v5.html_files/icons/go.png]

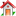

Supplement: Supplementary file 1 [file resprot_v3i1e4_app1.zip › Interactive thematic map/thematic map v5.html_files/icons/gohome.png]

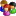

Supplement: Supplementary file 1 [file resprot_v3i1e4_app1.zip › Interactive thematic map/thematic map v5.html_files/icons/group.png]

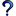

Supplement: Supplementary file 1 [file resprot_v3i1e4_app1.zip › Interactive thematic map/thematic map v5.html_files/icons/help.png]

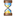

Supplement: Supplementary file 1 [file resprot_v3i1e4_app1.zip › Interactive thematic map/thematic map v5.html_files/icons/hourglass.png]

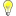

Supplement: Supplementary file 1 [file resprot_v3i1e4_app1.zip › Interactive thematic map/thematic map v5.html_files/icons/idea.png]

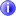

Supplement: Supplementary file 1 [file resprot_v3i1e4_app1.zip › Interactive thematic map/thematic map v5.html_files/icons/info.png]

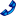

Supplement: Supplementary file 1 [file resprot_v3i1e4_app1.zip › Interactive thematic map/thematic map v5.html_files/icons/kaddressbook.png]

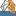

Supplement: Supplementary file 1 [file resprot_v3i1e4_app1.zip › Interactive thematic map/thematic map v5.html_files/icons/kmail.png]

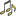

Supplement: Supplementary file 1 [file resprot_v3i1e4_app1.zip › Interactive thematic map/thematic map v5.html_files/icons/knotify.png]

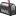

Supplement: Supplementary file 1 [file resprot_v3i1e4_app1.zip › Interactive thematic map/thematic map v5.html_files/icons/korn.png]

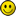

Supplement: Supplementary file 1 [file resprot_v3i1e4_app1.zip › Interactive thematic map/thematic map v5.html_files/icons/ksmiletris.png]

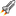

Supplement: Supplementary file 1 [file resprot_v3i1e4_app1.zip › Interactive thematic map/thematic map v5.html_files/icons/launch.png]

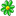

Supplement: Supplementary file 1 [file resprot_v3i1e4_app1.zip › Interactive thematic map/thematic map v5.html_files/icons/licq.png]

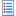

Supplement: Supplementary file 1 [file resprot_v3i1e4_app1.zip › Interactive thematic map/thematic map v5.html_files/icons/list.png]

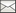

Supplement: Supplementary file 1 [file resprot_v3i1e4_app1.zip › Interactive thematic map/thematic map v5.html_files/icons/Mail.png]

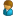

Supplement: Supplementary file 1 [file resprot_v3i1e4_app1.zip › Interactive thematic map/thematic map v5.html_files/icons/male1.png]

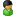

Supplement: Supplementary file 1 [file resprot_v3i1e4_app1.zip › Interactive thematic map/thematic map v5.html_files/icons/male2.png]

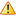

Supplement: Supplementary file 1 [file resprot_v3i1e4_app1.zip › Interactive thematic map/thematic map v5.html_files/icons/messagebox_warning.png]

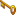

Supplement: Supplementary file 1 [file resprot_v3i1e4_app1.zip › Interactive thematic map/thematic map v5.html_files/icons/password.png]

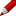

Supplement: Supplementary file 1 [file resprot_v3i1e4_app1.zip › Interactive thematic map/thematic map v5.html_files/icons/pencil.png]

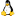

Supplement: Supplementary file 1 [file resprot_v3i1e4_app1.zip › Interactive thematic map/thematic map v5.html_files/icons/penguin.png]

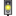

Supplement: Supplementary file 1 [file resprot_v3i1e4_app1.zip › Interactive thematic map/thematic map v5.html_files/icons/prepare.png]

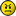

Supplement: Supplementary file 1 [file resprot_v3i1e4_app1.zip › Interactive thematic map/thematic map v5.html_files/icons/smiley-angry.png]

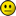

Supplement: Supplementary file 1 [file resprot_v3i1e4_app1.zip › Interactive thematic map/thematic map v5.html_files/icons/smiley-neutral.png]

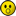

Supplement: Supplementary file 1 [file resprot_v3i1e4_app1.zip › Interactive thematic map/thematic map v5.html_files/icons/smiley-oh.png]

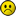

Supplement: Supplementary file 1 [file resprot_v3i1e4_app1.zip › Interactive thematic map/thematic map v5.html_files/icons/smily_bad.png]

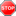

Supplement: Supplementary file 1 [file resprot_v3i1e4_app1.zip › Interactive thematic map/thematic map v5.html_files/icons/stop-sign.png]

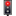

Supplement: Supplementary file 1 [file resprot_v3i1e4_app1.zip › Interactive thematic map/thematic map v5.html_files/icons/stop.png]

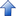

Supplement: Supplementary file 1 [file resprot_v3i1e4_app1.zip › Interactive thematic map/thematic map v5.html_files/icons/up.png]

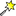

Supplement: Supplementary file 1 [file resprot_v3i1e4_app1.zip › Interactive thematic map/thematic map v5.html_files/icons/wizard.png]

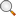

Supplement: Supplementary file 1 [file resprot_v3i1e4_app1.zip › Interactive thematic map/thematic map v5.html_files/icons/xmag.png]

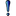

Supplement: Supplementary file 1 [file resprot_v3i1e4_app1.zip › Interactive thematic map/thematic map v5.html_files/icons/yes.png]

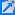

Supplement: Supplementary file 1 [file resprot_v3i1e4_app1.zip › Interactive thematic map/thematic map v5.html_files/ilink.png]

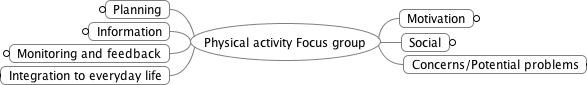

Supplement: Supplementary file 1 [file resprot_v3i1e4_app1.zip › Interactive thematic map/thematic map v5.html_files/image.png]

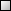

Supplement: Supplementary file 1 [file resprot_v3i1e4_app1.zip › Interactive thematic map/thematic map v5.html_files/leaf.png]

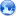

Supplement: Supplementary file 1 [file resprot_v3i1e4_app1.zip › Interactive thematic map/thematic map v5.html_files/map_location.png]

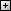

Supplement: Supplementary file 1 [file resprot_v3i1e4_app1.zip › Interactive thematic map/thematic map v5.html_files/show.png]
